# Is there a role for early postnatal steroids in very preterm infants exposed to chorioamnionitis?

**DOI:** 10.1038/s41390-024-03031-8

**Published:** 2024-01-20

**Authors:** Viral G. Jain, Namasivayam Ambalavanan

**Affiliations:** Division of Neonatology, Department of Pediatrics, University of Alabama at Birmingham, Birmingham, AL USA

Acute chorioamnionitis is one of the most common causes of very preterm birth, thereby increasing the risk of adverse neonatal outcomes, including bronchopulmonary dysplasia (BPD) and neurodevelopmental impairment (NDI).^1^ Despite recent advancements in neonatal care, the rates of BPD have increased for very preterm neonates, especially in those exposed to chorioamnionitis.^2,3^ The inflammation associated with chorioamnionitis is considered to be the primary driver of damage to developing neonatal organs.^4^ Thus, a better understanding of therapeutic strategies to reduce such inflammation is needed for preventing chorioamnionitis-associated BPD.

Early (≤7 days) moderate-to-high-dose postnatal dexamethasone for ventilator-dependent preterm infants decreases lung inflammation and facilitates extubation, thereby reducing the risk of BPD.^5^ However, it is also associated with many short-term adverse effects, including intestinal perforation and growth failure. An increased risk of NDI, particularly cerebral palsy, was noted with early dexamethasone, which led to declining use of such a strategy. Is it possible that a subset of preterm infants may benefit from early postnatal steroid therapy?

In this issue, Papagianis et al.^6^ chose a pragmatic approach using a low-dose taper of dexamethasone similar to the DART trial,^7^ which is widely used for evolving and established BPD (>8 days) to facilitate extubation. Papagianis and colleagues investigated whether low-dose dexamethasone administered soon after birth in acute prenatal inflammation-exposed preterm sheep would reduce ventilator requirements and prevent inflammation and BPD-like lung pathology. Pregnant sheep were given intra-amniotic lipopolysaccharide (LPS) or saline injections to induce antenatal inflammation. The lambs were delivered 24–48 h later via C-section, resuscitated, given surfactant, and mechanically ventilated. Respiratory support was weaned as per established criteria. They found that antenatal inflammation-exposed lambs who received postnatal steroids had thinner lung tissue, increased airspace, and reduced inflammatory and proliferative cells within the lungs compared to those without steroid exposure at 7 days after delivery. However, no change in the duration of mechanical ventilation or blood gases was noted.

This study suggest that there is possibly a beneficial role of steroids in chorioamnionitis-exposed infants. In preterm sheep and rhesus macaque model of chorioamnionitis, antenatal inflammation induced lung maturation, improved lung compliance, and increased surfactant production. Antenatal steroids further enhanced lung maturation relative to antenatal inflammation or steroid exposure alone.^8,9^ This is also consistent with studies in preterm infants exposed to chorioamnionitis that have shown that antenatal steroids reduce mortality and respiratory distress syndrome (RDS) but not BPD.^10^ Chorioamnionitis decreases the magnitude of RDS but increases the risk of BPD.^4,11^ Steroids accelerate lung maturation but inhibit proliferation/growth and thus may increase the risk of BPD if infants continue to be exposed to hyperoxia and volutrauma due to mechanical ventilation. Therefore, cautious use of early postnatal steroids is warranted, as an early respiratory benefit and reduction in BPD may be counterbalanced by a higher risk of NDI and possibly no reduction in BPD if the infant continues to require mechanical ventilation and high FiO_2_. Based on the data by Papagianis and colleagues, is there a way forward to using postnatal steroids in chorioamnionitis-exposed infants?

Steroid-induced neuronal injury may depend on the relative effects of glucocorticoid receptor versus mineralocorticoid receptor activity. Glucocorticoid activity is linked to apoptosis, while mineralocorticoid stimulation protects from neuronal injury.^12^ Dexamethasone has only glucocorticoid effects with no mineralocorticoid activity. Meanwhile, hydrocortisone activates both the glucocorticoid and the mineralocorticoid receptors in a less potent manner.^13^ Studies have shown the positive effect of early low-dose hydrocortisone on neonatal mortality and survival free of BPD in chorioamnionitis-exposed infants.^14,15^ These findings suggest that early low-dose hydrocortisone may be worthwhile to explore in chorioamnionitis-exposed preterm neonates and compare with dexamethasone. It is important to remember that BPD is a clinical operational definition and does not specify pathophysiology—infants have varying combinations of inhibition of lung alveolar development, microvascular development, airway malacia, lung fibrosis, inflammation, and abnormal control of breathing. It is therefore necessary to carefully define and characterize short- and long-term respiratory outcomes as well as non-respiratory outcomes such as neurodevelopment in preclinical models and subsequently in human preterm infants in clinical trials.
